# 液液萃取-气相色谱-质谱法同时测定水中46种半挥发性有机物

**DOI:** 10.3724/SP.J.1123.2020.07006

**Published:** 2021-05-08

**Authors:** Lingling LIU, Lijun ZHANG, Xiliang DONG, Xiaomei CHEN, Chuanming ZHAO

**Affiliations:** 济南市环境研究院, 山东 济南 250100; Jinan Environmental Research Institute, Jinan 250100, China; 济南市环境研究院, 山东 济南 250100; Jinan Environmental Research Institute, Jinan 250100, China; 济南市环境研究院, 山东 济南 250100; Jinan Environmental Research Institute, Jinan 250100, China; 济南市环境研究院, 山东 济南 250100; Jinan Environmental Research Institute, Jinan 250100, China; 济南市环境研究院, 山东 济南 250100; Jinan Environmental Research Institute, Jinan 250100, China

**Keywords:** 气相色谱-质谱, 液液萃取, 半挥发性有机物, gas chromatography-mass spectrometry (GC-MS), liquid-liquid extraction (LLE), semi-volatile organic compounds (SVOCs)

## Abstract

半挥发性有机物主要包括多环芳烃类(PAHs)、邻苯二甲酸酯类(PAEs)、有机氯农药类(OCPs)和硝基苯类(NBs)等化合物,这些物质多具有致癌、致畸、致突变作用,以及内分泌干扰效应。因此,快速准确测定水中半挥发性有机物非常重要,目前国内尚无水中半挥发性有机物的检测标准。该研究从氮吹温度、水样pH值和萃取时间3个方面进行了优化,旨在建立一种液液萃取-气相色谱-质谱(LLE-GC-MS)同时测定水中46种半挥发性有机物的方法。结果表明:氮吹温度对46种半挥发性有机物的回收率影响不大,考虑回收率及浓缩效率,将氮吹温度设定为35 ℃;水样在中性环境下萃取效果好于碱性环境下的效果;萃取时间由7 min增加至10 min时,回收率也随之提高,但时间增加至15 min时,17种(占比37%)化合物回收率有所增加,29种(占比63%)化合物回收率则呈降低趋势。因此,将萃取时间设定为每次10 min。采用气相色谱-质谱仪进行检测,内标法定量。该方法在20.0~2000 μg/L范围内线性良好,相关系数(*r* ^2^)≥0.9916, 46种SVOCs检出限为0.28~16.55 ng/L,定量限为0.92~55.16 ng/L;在0.02、0.2、0.4 μg/L 3个加标水平下的平均回收率为63.6%~125%,相对标准偏差(*n*=6)为1.03%~17.0%。采用该方法检测了黄河流域济南段的27个地表水样品,检出的物质以PAEs和PAHs为主,2种OCPs在部分点位有检出,NBs均未检出。该方法操作简单,通用性强,准确度及精密度良好,检出限低,适用于地表水及地下水中46种半挥发性有机物的同时检测。

## Abstract (en)

Semi-volatile organic compounds (SVOCs) include polycyclic aromatic hydrocarbons (PAHs), phthalic acid esters (PAEs), organochlorine pesticides (OCPs) and nitrobenzenes (NBs). Most of them have carcinogenic, teratogenic, mutagenic and endocrine disrupting effects. Therefore, rapid and accurate determination of SVOCs in water is very important. As the most traditional pretreatment method, liquid-liquid extraction (LLE) has the advantages of wide extraction range, high extraction efficiency, simple operation and lower cost, which is very suitable for the simultaneous extraction of multiclass SVOCs. Dichloromethane has good solubility for most SVOCs, and is slightly soluble in water with low boiling point. It is a good broad-spectrum extractive solvent of organic compounds. But at present, there is no detection standard of SVOCs in water in China. In this study, three factors including nitrogen blowing temperature, pH of water sample and extraction time were optimized. It was aimed to establish a liquid-liquid extraction-gas chromatography-mass spectrometry (GC-MS) method for the simultaneous determination of the 46 SVOCs in water. At first, the effect of nitrogen blowing temperature (30, 35, 40 ℃) was investigated. The results showed that under different nitrogen blowing temperature, the recoveries of the 46 SVOCs were slightly different, but the differences were not significant. Considering the recovery and concentration efficiency, the nitrogen blowing temperature was finally set at 35 ℃. Dichloromethane was selected as the LLE solvent of the SVOCs and its extraction efficiency was investigated. The recoveries of the 46 SVOCs were satisfactory for the determination. Then sample pH (neutral and alkaline condition) was investigated. Most of the SVOCs in this paper have no obvious acid-base property. The extraction effect of water sample under neutral conditions was the best and the most stable, and under alkaline condition, the recovery of each substance was generally low. Finally, extraction time (7, 10, 15 min) was studied. In a certain range, with the increase of extraction time, the recovery also increased, but when the time increased to 15 min, the recovery of some compounds increased or decreased, and the time-consuming was longer, the recovery of most substances could meet the requirements when the extraction time was set to 10 min. The optimized experimental conditions were determined as follows: under neutral conditions, the water sample was extracted by dichloromethane for three times, each extraction time was 10 min, and concentrated at the nitrogen blowing temperature of 35 ℃. GC-MS was used for detection, and internal standard method was used for quantitative analysis. The results showed that the linearity of the method was good in the range of 20-2000 μg/L, the correlation coefficients (*r* ^2^) were no less than 0.9916, the limits of detection (LODs, *S/N*=3) ranged from 0.28 to 16.55 ng/L, and the limits of quantification (LOQs, *S/N*=10) ranged from 0.92 to 55.16 ng/L. The average recovery was 63.6%-125% at three spiked levels of 0.02, 0.2, 0.4 μg/L, with the relative standard deviations (*n*=6) ranging from 1.03% to 17.0%. This method was applied for the determination of 27 surface water samples in Jinan section of the Yellow River. The pollutants were mainly PAEs and PAHs, while NBs were not detected, only two kinds of OCPs were detected at some sites. The method is simple, universal, accurate and precise, and has low detection limit. It is suitable for the simultaneous determination of the 46 SVOCs in surface water and groundwater.

半挥发性有机物(SVOCs),一般是指沸点范围为170~350 ℃的有机物,主要包括多环芳烃类(PAHs)、邻苯二甲酸酯类(PAEs)、有机氯农药类(OCPs)和硝基苯类(NBs)等化合物^[1]^。这些物质多具有致癌、致畸、致突变作用,以及内分泌干扰效应和生殖发育毒性,严重危害人类健康和生态环境^[2]^。我国环保部和美国国家环境保护局(EPA)已将20多种半挥发性有机物列入优先控制的污染物名单中^[1]^。

SVOCs的前处理方法主要有液液萃取^[2,3]^、固相萃取^[1,4,5]^和固相微萃取^[6]^。固相萃取和固相微萃取技术由于萃取填料或涂层仅对特定化合物有选择性吸附,较难同时准确测定不同类别的SVOCs,且成本较高^[2]^。而液液萃取作为最传统的前处理方法,具有萃取范围广、萃取效率高、操作简单、成本低廉等突出优点,非常适合多类SVOCs的同时萃取^[2]^。

常用的检测方法有气相色谱法(GC)、气相色谱-质谱法(GC-MS)、气相色谱-串联质谱法(GC-MS/MS)。GC选择性强,灵敏度高,但仅靠保留时间定性,容易出现假阳性现象。GC-MS/MS定性能力强,检出限低,但价格昂贵,推广性不强。与其他两种检测方法相比,GC-MS具有较强的定性能力和较低的价格,因此应用范围较广。

目前国内尚无水中多种SVOCs的相关检测标准,仅在《水和废水监测分析方法》(第四版)(增补版)^[7]^中可见半挥发性有机物的测定方法,但该方法为C类方法,尚未经多个实验室认证。且该方法SVOCs的回收率波动性太大(D~262%, D为有检出,且结果大于0),并不适用于所有SVOCs的检测,方法仍需改进。目前黄河流域济南段地表水中SVOCs的污染水平鲜见报道。国内研究者对水中NBs^[8,9]^、OCPs^[10,11]^、PAHs^[12,13,14]^和PAEs^[15,16,17]^或某2类^[18,19,20,21]^及3类以上^[1-3,6]^检测也多有研究,但同时测定2种硝基苯、16种多环芳烃、23种有机氯和5种邻苯二甲酸酯类化合物的研究甚少。

本研究通过优化氮吹温度、水样pH值、萃取时间等前处理条件,建立了液液萃取结合气相色谱-质谱同时测定水中46种SVOCs的方法,并运用该方法对黄河流域济南段地表水进行了检测,为了解该流域地表水污染水平提供了参考。该方法具有灵敏度高、重复性好、操作简单和通用性强等优点,可为环境水体中多类SVOCs的同时检测提供有力的技术支持。

## 1 实验部分

### 1.1 仪器、试剂与材料

7890B/5977B气相色谱-质谱仪、HP-5MS(30 m×0.25 mm×0.25 μm)毛细管柱(美国安捷伦公司); M10平行蒸发浓缩仪(北京莱伯泰科公司); JW-C分液漏斗振荡器(常州顶新公司); Milli-Q超纯水机(德国默克公司)。

正己烷、丙酮、二氯甲烷均为色谱纯(美国Tedia公司产品);无水硫酸钠(分析纯)、氯化钠(优级纯)(国药公司产品);实验用超纯水(Milli-Q超纯水机自制)。

23种有机氯农药混合标准溶液(1000 μg/mL,溶剂为甲苯-正己烷(1∶1, v/v))、硝基苯(1000 μg/mL,溶剂为甲醇)均为美国AccuStandard公司产品;16种多环芳烃混合标准溶液(2000 μg/mL,溶剂为甲苯-二氯甲烷(1∶1, v/v))、5种邻苯二甲酸酯混合标准溶液(2000 μg/mL,溶剂为正己烷)、2,4-二硝基甲苯(1000 μg/mL,溶剂为乙腈)均为美国O2si公司产品。

5种替代物(SS):硝基苯-d_5_(2000 μg/mL,溶剂为二氯甲烷)、2-氟联苯和4,4'-三联苯-d_14_混合标准溶液(2000 μg/mL,溶剂为丙酮-正己烷(1∶1, v/v))、四氯间二甲苯和十氯联苯混合标准溶液(2000 μg/mL,溶剂为甲苯-正己烷(1∶1, v/v)),以及5种内标(IS):萘-d_8_、苊-d_10_、菲-d_10_、䓛-d_12_、苝-d_12_(2000 μg/mL,溶剂为二氯甲烷)均为美国O2si公司产品。

### 1.2 样品前处理

取1 L水样,置于2 L分液漏斗中,加入30 g氯化钠(使有机相与水相更好的分离),摇匀,加入50 mL二氯甲烷,轻轻摇匀并注意放气,在分液漏斗振荡器上振荡萃取10 min,静置分层10 min,下层有机相经装有无水硫酸钠(预先用二氯甲烷润洗)的砂芯漏斗进行脱水,接收至200 mL的浓缩杯中,重复上述步骤2次,收集所有萃取液,氮吹浓缩至1 mL以下,加入20 μL 40 μg/mL的内标中间液,用正己烷定容至1 mL,上机待测。

### 1.3 分析条件

1.3.1 色谱条件

色谱柱:HP-5MS柱(30 m×0.25 mm×0.25 μm);进样口温度:280 ℃,载气(He)流速:1.0 mL/min,升温程序:50 ℃保持1 min,以10 ℃/min升至150 ℃,保持5 min,再以3 ℃/min升至290 ℃,保持1 min(运行时间63.667 min);进样量:1.0 μL;不分流进样。

1.3.2 质谱条件

离子源:电子轰击(EI)源;电子能量:70 eV;传输线温度:280 ℃;离子源温度:230 ℃;四极杆温度:150 ℃;溶剂延迟时间:6 min;扫描方式:选择离子扫描模式。46种SVOCs、5种替代物、5种内标的保留时间、定量离子和定性离子见表1。

**表 1:** 46种半挥发性有机物、5种替代物和5种内标的保留时间、定量离子和定性离子

No.	Compound	Abbreviation	*t*_R_/ min	Quantitative ion (*m/z*)	Qualitative ions (*m/z*)		IS		Type
1	nitrobenzene (硝基苯)	NB	7.93	77	51/123	NAP-d_8_		NBs	
2	naphthalene (萘)	NAP	9.43	128	129	NAP-d_8_		PAHs	
3	dimethyl phthalate (邻苯二甲酸二甲酯)	DMP	13.68	163	77	ACE-d_10_		PAEs	
4	acenaphthylene (苊烯)	ACY	13.70	152	151/153	ACE-d_10_		PAHs	
5	acenaphthene (苊)	ACE	14.50	153	152/154	ACE-d_10_		PAHs	
6	2,4-dinitrotoluene (2,4-二硝基甲苯)	2,4-DNT	15.73	165	63/89	ACE-d_10_		NBs	
7	fluorene (芴)	FL	17.27	166	82/163	ACE-d_10_		PAHs	
8	diethyl phthalate (邻苯二甲酸二乙酯)	DEP	17.59	149	177	ACE-d_10_		PAEs	
9	*α*-benzenehexachloride (*α*-六六六)	*α*-BHC	20.99	181	109/183	PHE-d_10_		OCPs	
10	hexachlorobenzene (六氯苯)	HCB	21.40	284	282/286	PHE-d_10_		OCPs	
11	*β*-benzenehexachloride (*β*-六六六)	*β*-BHC	22.75	181	109/183	PHE-d_10_		OCPs	
12	*γ*-benzenehexachloride (*γ*-六六六)	*γ*-BHC	23.09	181	109/183	PHE-d_10_		OCPs	
13	phenanthrene (菲)	PHE	23.58	178	176/179	PHE-d_10_		PAHs	
14	anthracene (蒽)	ANT	23.89	178	176/179	PHE-d_10_		PAHs	
15	*δ*-benzenehexachloride (*δ*-六六六)	*δ*-BHC	24.69	181	109/183	PHE-d_10_		OCPs	
16	heptachlor (七氯)	/	27.60	100	272/274	PHE-d_10_		OCPs	
17	aldrin (艾氏剂)	/	29.72	66	220/263	PHE-d_10_		OCPs	
18	di-*n*-butyl phthalate (邻苯二甲酸二正丁酯)	D(*n*)BP	29.76	149	76/150	PHE-d_10_		PAEs	
19	heptachlor epoxide (环氧化七氯)	/	32.31	353	351/355	CHR-d_12_		OCPs	
20	fluoranthene (荧蒽)	FLU	32.40	202	200/203	CHR-d_12_		PAHs	
21	*α*-chlordane (*α*-氯丹)	/	33.78	373	375/377	CHR-d_12_		OCPs	
22	pyrene (芘)	PYR	33.94	202	200/203	CHR-d_12_		PAHs	
23	*α*-endosulfan (*α*-硫丹)	/	34.47	195	339/341	CHR-d_12_		OCPs	
24	*γ*-chlordane (*γ*-氯丹)	/	34.72	375	237/272	CHR-d_12_		OCPs	
25	dieldrin (狄氏剂)	/	36.06	79	263/279	CHR-d_12_		OCPs	
26	*p*,*p*'-1,1-dichloro-2,2-bis(4-chorophenyl)ethylene (*p*,*p*'-滴滴伊)	*p*,*p*'-DDE	36.31	246	176/248	CHR-d_12_		OCPs	
27	endrin (异狄氏剂)	/	37.32	263	81/82	CHR-d_12_		OCPs	
28	*β*-endosulfan (*β*-硫丹)	/	37.93	195	207/339	CHR-d_12_		OCPs	
29	*p*,*p*'- bis(4-chlorophenyl)-1,1-dichloroethane (*p*,*p*'-滴滴滴)	*p*,*p*'-DDD	38.81	235	165/237	CHR-d_12_		OCPs	
30	*o*,*p*'-dichlorodiphenyltrichloroethane (*o*,*p*'-滴滴涕)	*o*,*p*'-DDT	38.95	235	165/237	CHR-d_12_		OCPs	
31	endrin aldehyde (异狄氏剂醛)	/	39.13	67	250/345	CHR-d_12_		OCPs	
32	endosulfan sulfate (硫丹硫酸酯)	/	40.55	272	387/422	CHR-d_12_		OCPs	
33	*p*,*p*'-dichlorodiphenyltrichloroethane (*p*,*p*'-滴滴涕)	*p*,*p*'-DDT	41.01	235	165/237	CHR-d_12_		OCPs	
34	benzyl butyl phthalate (邻苯二甲酸丁基苄基酯)	BBP	41.09	149	91/206	CHR-d_12_		PAEs	
35	endrin ketone (异狄氏剂酮)	/	43.16	317	67/147	CHR-d_12_		OCPs	
36	benzo[*a*]anthracene (苯并[*a*]蒽)	BaA	43.33	228	226/229	CHR-d_12_		PAHs	
37	chrysene ($\overset{艹}{屈}$)	CHR	43.61	228	226/229	CHR-d_12_		PAHs	
38	methoxychlor (甲氧滴滴涕)	/	44.61	227	152/228	CHR-d_12_		OCPs	
39	mirex (灭蚁灵)	/	46.25	272	270/274	CHR-d_12_		OCPs	
40	di-*n*-octyl phthalate (邻苯二甲酸二正辛酯)	D(*n*)OP	50.88	149	279	PER-d_12_		PAEs	
41	benzo[*b*]fluoranthene (苯并[*b*]荧蒽)	BbF	51.07	252	126/250	PER-d_12_		PAHs	
42	benzo[*k*]fluoranthene (苯并[*k*]荧蒽)	BkF	51.23	252	126/250	PER-d_12_		PAHs	
43	benzo[*a*]pyrene (苯并[*a*]芘)	BaP	53.05	252	250/253	PER-d_12_		PAHs	
44	indeno[1,2,3-cd]pyrene (茚并(1,2,3-cd)芘)	InP	59.80	276	138/274	PER-d_12_		PAHs	
45	dibenzo[*a*,*h*]anthracene (二苯并[*a*,*h*]蒽)	DahA	60.13	278	139/276	PER-d_12_		PAHs	
46	benzo[*g*,*h*,*i*]perylene (苯并[*g*,*h*,*i*]芘)	BghiP	61.08	276	138/274	PER-d_12_		PAHs	
SS1	nitrobenzene-d_5_ (硝基苯-d_5_)	NB-d_5_	7.90	82	54/128	NAP-d_8_		SS	
SS2	2-fluorobiphenyl (2-氟联苯)	/	12.07	172	170/171	NAP-d_8_		SS	
SS3	2,4,5,6-tetrachloro-*m*-xylene (四氯间二甲苯)	/	18.41	207	242/244	PHE-d_10_		SS	
SS4	*p*-terphenyl-d_14_ (4,4'-三联苯-d_14_)	/	36.25	244	243/245	CHR-d_12_		SS	
No.	Compound	Abbreviation	*t*_R_/ min	Quantitative ion (*m/z*)	Qualitative ions (*m/z*)		IS		Type
SS5	decachlorobiphenyl (十氯联苯)	PCB209	54.10	498	178/214	PHE-d_10_		SS	
IS1	naphthalene-d_8_ (萘-d_8_)	NAP-d_8_	9.39	136	108			IS	
IS2	acenaphthene-d_10_(苊-d_10_)	ACE-d_10_	14.38	164	160/162			IS	
IS3	phenanthrene-d_10_ (菲-d_10_)	PHE-d_10_	23.44	188	160/189			IS	
IS4	chrysene-d_12_ ($\overset{艹}{屈}$-d_12_)	CHR-d_12_	43.45	240	236/241			IS	
IS5	perylene-d_12_(苝-d_12_)	PER-d_12_	53.47	264	263/260			IS	

PAHs: polycyclic aromatic hydrocarbons; OCPs: organochlorine pesticides; PAEs: phthalic acid esters.

### 1.4 质控措施

1.4.1 空白干扰的控制

因有机物特别是邻苯二甲酸酯类极易受到空白干扰,实验室空气及实验过程中用到的试剂、器皿均会引入干扰,因此,须采取严格的质控措施降低空白干扰。如实验用到的氯化钠、无水硫酸钠均经过600 ℃高温烘烤4 h;所有用到的玻璃器皿及无水硫酸钠在使用前均用二氯甲烷润洗2遍;实验过程中尽量避免使用塑料制品,以将空白干扰降到最低。

1.4.2 替代物回收率控制

选用与目标物性质相似、保留时间接近且样品中不存在的物质作为替代物,在样品提取之前加入,目标物在样品前处理过程中的回收率可通过已知的替代物回收率来衡量。

## 2 结果与讨论

### 2.1 色谱-质谱条件的优化

本实验采用HP-5MS毛细管色谱柱对目标物进行分离,分别通过全扫描(Full Scan)和选择离子监测(SIM)模式进行定性和定量测定,选择丰度高、干扰低的特征离子为定量离子,丰度较高、干扰较低的1个或2个特征离子作为定性离子。46种SVOCs混合标准溶液(2000 μg/L)、5种替代物(2000 μg/L)和5种内标(800 μg/L)的总离子流色谱图见图1。由图1可知,虽然部分化合物如硝基苯和硝基苯-d_5_,萘和萘-d_8_未能完全实现基线分离,但因其定量和定性离子不同,因此不影响分析。

**图 1:**
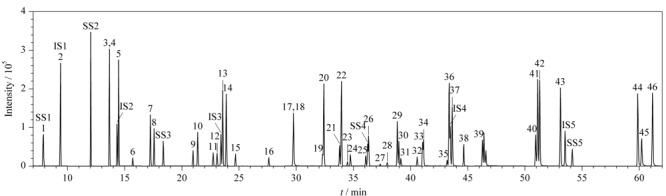
46种SVOCs(2000 μg/L)、5种替代物(2000 μg/L)及5种内标(800 μg/L)的TIC色谱图

### 2.2 前处理条件的优化

2.2.1 氮吹温度的影响

氮吹时的温度是影响回收率的重要因素,特别是对沸点较低的化合物影响较大。在150 mL二氯甲烷中加入0.2 μg/L的SVOCs混合标准溶液,分别考察了氮吹温度为30、35、40 ℃时各目标化合物的回收率(平行测定3次)。

在氮吹温度为30 ℃时,46种SVOCs的平均回收率为64.6%~104%,当氮吹温度增加至35 ℃时,各化合物的回收率略有提高,为65.8%~106%。但随着温度增加至40 ℃,大部分化合物的回收率开始呈下降趋势,回收率为63.4%~100%。从研究结果来看,虽然在不同的氮吹温度下,各目标物的回收率略有差异,但差异并不显著,综合考虑回收率及浓缩效率,本研究将氮吹温度设定为35 ℃。

2.2.2 萃取溶剂的选择

本文的研究对象均为弱极性化合物,二氯甲烷和正己烷为弱极性和非极性有机溶剂,根据相似相溶原理,常用于水中SVOCs的萃取。但正己烷密度比水小,萃取液位于水面上方,转移时较为复杂。而二氯甲烷对多数SVOCs具有良好的溶解性,且微溶于水,沸点低(39 ℃),密度比水大,萃取液位于水面下方,转移方便,是良好的广谱性有机物萃取剂,被US EPA 3510C等方法选用^[2]^。因此,本文选择二氯甲烷作为SVOCs液液萃取剂,并考察了其萃取效率。结果表明,二氯甲烷作为萃取剂时,46种SVOCs的回收率为69.5%~121%,可满足大部分SVOCs的萃取要求。

2.2.3 水样pH值的影响

为了考察液液萃取时水样pH值对萃取效果的影响,本研究参考《水和废水监测分析方法》(第四版)(增补版)^[7]^中半挥发性有机物的测定方法及前人的研究成果,分别在碱性(pH>12)和中性(6<pH<9)条件下,对加标量0.2 μg/L的水样进行分析,平行测定3次。结果表明,在碱性条件下,各物质的回收率普遍偏低,为33.7%~102%,其中15种(占比33%)化合物的回收率小于60%。而在中性条件下,所有化合物的回收率均大于60%,为61.1%~129%,基本满足分析要求。分析原因可能是NBs、OCPs、PAHs及PAEs均无明显的酸碱性,因此在水样pH值为中性时,萃取效果最好而且最稳定。这与前人的研究结果一致^[5,10]^。

2.2.4 萃取时间的影响

振荡萃取时间是影响回收率的重要因素,本研究分别考察了振荡7、10和15 min时的回收率(见图2)。结果表明,萃取时间由7 min增至10 min时,回收率明显增加。但萃取时间继续增加至15 min时,17种(占比37%)化合物的回收率有所增加,29种(占比63%)化合物的回收率则呈现降低的趋势。综合考虑回收率及萃取时间,本实验将萃取时间设定为10 min。

**图 2:**
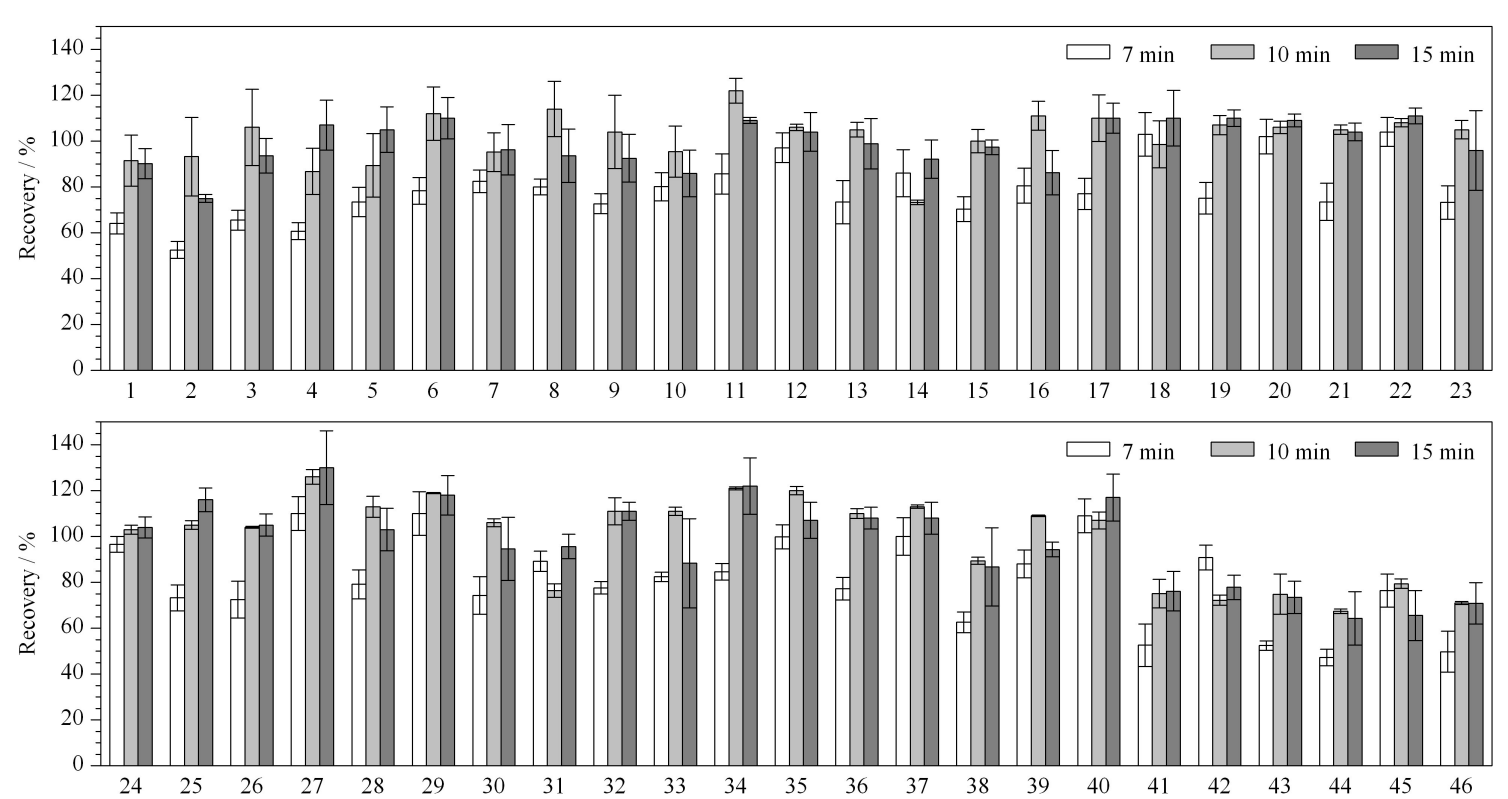
不同萃取时间对46种SVOCs回收率的影响(*n*=3)

### 2.3 方法学考察

2.3.1 线性范围及检出限

配制质量浓度为20.0、60.0、100、200、400、800、2000 μg/L的46种SVOCs及5种替代物混合标准溶液,以目标化合物与内标的峰面积比值对其相应的质量浓度进行线性回归,绘制标准工作曲线(见表2)。结果表明,在20.0~2000 μg/L的线性范围内,46种SVOCs及5种SSs呈较好的线性关系,相关系数(*r*^2^)≥0.9916。

**表 2:** 46种SVOCs及5种替代物的线性方程、相关系数、检出限、定量限、加标回收率及相对标准偏差

Compound	*r* ^2^	Linear equation	LOD/ (ng/L)	LOQ/ (ng/L)	0.02 μg/L			0.2 μg/L			0.4 μg/L		
					Recovery/%	RSD/%		Recovery/%	RSD/%		Recovery/%		RSD/%
NB	0.9968	*y*=0.1478*x*	1.18	3.92	92.6	2.19		96.7	13.1		97.3		8.83
NAP	0.9997	*y*=0.8037*x*	0.36	1.20	93.1	2.79		95.1	14.0		103		6.58
DMP	0.9988	*y*=0.8904*x*	0.32	1.05	105	7.03		109	14.7		106		7.99
ACY	0.9995	*y*=1.5014*x*	0.28	0.92	89.0	1.03		88.4	12.5		80.7		4.85
ACE	0.9993	*y*=1.0285*x*	0.35	1.17	91.5	2.58		91.1	12.9		100		7.36
2,4-DNT	0.9921	*y*=0.1181*x*	4.55	15.17	112	7.09		113	12.4		115		5.05
FL	0.9987	*y*=1.2091*x*	0.72	2.41	99.3	2.56		96.5	7.67		107		5.71
DEP	0.9979	*y*=0.8836*x*	0.99	3.29	111	3.05		116	10.1		113		4.48
*α*-BHC	0.9986	*y*=0.0931*x*	2.39	7.98	101	2.59		107	14.5		110		2.53
HCB	0.9998	*y*=0.2157*x*	1.09	3.65	96.3	3.62		96.2	10.2		103		3.92
*β*-BHC	0.9976	*y*=0.0705*x*	2.74	9.14	125	4.41		122	5.30		117		3.70
*γ*-BHC	0.9979	*y*=0.0757*x*	2.82	9.41	113	2.96		108	7.44		107		8.26
PHE	0.9996	*y*=0.8830*x*	0.49	1.63	111	3.84		110	9.14		110		5.09
ANT	0.9987	*y*=0.8224*x*	0.52	1.72	97.4	5.21		72.9	1.23		78.0		3.08
*δ*-BHC	0.9980	*y*=0.0721*x*	2.99	9.98	101	4.62		99.9	6.09		101		4.09
Heptachlor	0.9957	*y*=0.0516*x*	4.23	14.11	115	3.87		108	4.77		109		4.51
Aldrin	0.9990	*y*=0.0721*x*	0.70	2.32	118	1.46		114	8.67		114		3.78
D(*n*)BP	0.9933	*y*=0.7073*x*	2.09	6.97	99.5	9.19		97.4	8.70		110		3.81
Heptachlor epoxide	0.9965	*y*=0.0757*x*	3.08	10.26	103	6.24		104	8.51		106		3.10
FLU	0.9986	*y*=0.9876*x*	0.45	1.50	107	3.50		105	4.91		105		1.76
*α*-Chlordane	0.9976	*y*=0.1282*x*	1.78	5.94	102	5.78		102	6.69		100		3.25
PYR	0.9986	*y*=1.0056*x*	0.44	1.46	114	3.06		107	3.81		105		2.16
*α*-Endosulfan	0.9993	*y*=0.0213*x*	8.38	27.94	102	4.86		103	6.25		102		3.71
*γ*-Chlordane	0.9995	*y*=0.1028*x*	3.22	10.74	100	5.33		100	6.26		97.2		4.45
Dieldrin	0.9990	*y*=0.0806*x*	3.53	11.78	104	2.93		105	2.93		107		3.70
*p*,*p*'-DDE	0.9997	*y*=0.2042*x*	1.22	4.08	101	4.33		101	4.45		102		3.36
Endrin	0.9978	*y*=0.0123*x*	16.55	55.16	112	12.8		115	13.4		110		3.19
*β*-Endosulfan	0.9972	*y*=0.0231*x*	9.70	32.33	110	6.49		111	5.92		107		4.54
*p*,*p*'-DDD	0.9969	*y*=0.2911*x*	0.83	2.77	111	5.55		115	5.77		108		4.75
*o*,*p*'-DDT	0.9936	*y*=0.1616*x*	1.53	5.09	90.7	9.16		102	9.25		106		3.43
Endrin aldehyde	0.9988	*y*=0.0491*x*	4.91	16.35	87.7	6.75		78.3	8.40		82.4		5.43
Endosulfan sulfate	0.9968	*y*=0.0456*x*	4.01	13.36	116	4.99		114	6.76		114		3.79
*p*,*p*'-DDT	0.9938	*y*=0.1278*x*	1.56	5.19	97.1	9.88		107	8.66		108		3.91
BBP	0.9923	*y*=0.1936*x*	1.39	4.62	105	16.2		113	17.0		109		2.57
Endrin ketone	0.9964	*y*=0.0393*x*	6.02	20.07	114	5.17		116	5.44		108		3.77
BaA	0.9992	*y*=0.8580*x*	0.45	1.49	115	1.67		109	2.09		106		2.60
CHR	0.9998	*y*=0.5762*x*	0.64	2.12	116	4.75		112	1.52		106		3.36
Methoxychlor	0.9916	*y*=0.2006*x*	1.70	5.68	77.7	6.06		88.2	4.22		90.1		3.07
Mirex	0.9983	*y*=0.1595*x*	1.43	4.75	108	3.59		107	3.22		105		1.93
D(*n*)OP	0.9944	*y*=0.4437*x*	1.19	3.95	97.1	12.7		106	9.99		105		2.46
BbF	0.9986	*y*=0.9327*x*	0.43	1.43	71.3	5.50		76.2	5.87		77.1		2.23
BkF	0.9991	*y*=0.9372*x*	0.44	1.46	72.2	1.87		72.6	2.32		73.3		6.86
BaP	0.9991	*y*=0.8403*x*	0.47	1.58	87.6	5.41		71.1	11.6		81.2		6.69
InP	0.9947	*y*=0.8127*x*	0.51	1.70	63.6	6.10		71.0	6.92		84.2		2.77
DahA	0.9938	*y*=0.2950*x*	1.36	4.52	67.1	8.09		81.0	3.11		84.9		2.45
BghiP	0.9985	*y*=0.8231*x*	0.51	1.69	66.2	6.99		73.3	4.21		77.1		2.58
SS1	0.9974	*y*=0.1522*x*	/	/	93.4	2.61		101	10.6		101		8.84
SS2	0.9998	*y*=0.6873*x*	/	/	92.3	3.85		97.1	13.6		95.3		9.28
SS3	0.9921	*y*=0.1181*x*	/	/	89.6	4.24		96.9	17.0		98.1		4.92
SS4	0.9996	*y*=0.1550*x*	/	/	109	2.56		109	3.14		111		3.09
SS5	0.9995	*y*=0.1566*x*	/	/	76.9	5.91		76.2	4.98		76.3		5.97

*y*: peak area ratio of target compound to internal standard; *x*: content ratio of target compound to internal standard.

以信噪比(*S/N*)为3和10确定检出限(LOD)和定量限(LOQ)。本研究46种SVOCs的LOD和LOQ分别为0.28~16.55 ng/L和0.92~55.16 ng/L。

2.3.2 准确度和精密度

准确量取1 L自来水样品,添加0.02、0.2、0.4 μg/L 3个水平的46种SVOCs及5种替代物混合标准溶液,按照优化后的实验条件进行测定,每个加标水平平行测定6次,计算SVOCs的回收率及相对标准偏差,结果见表2。3个加标水平下46种SVOCs的平均加标回收率为63.6%~125%, RSD为1.03%~17.0%, 5种SSs的回收率为76.2%~111%,RSD为2.56%~17.0%,均基本满足检测要求。

### 2.4 实际样品的测定

采用本文建立的方法对黄河流域济南段的27个地表水样品进行检测。样品提取前,在实际样品中均添加10 μL 20 μg/mL的替代物混合标准溶液,以替代物的回收率评价目标物的回收率。结果表明,5种替代物的回收率为73.8%~109%,说明此次样品检测过程中目标物回收率可满足要求。27个样品中检出的物质以PAEs和PAHs为主,NBs在所有点位均未检出,OCPs除*α*-硫丹和灭蚁灵有检出外(含量为1.51~57.94 ng/L),其他有机氯农药均未检出。PAEs中DEP、BBP、D(*n*)OP均有不同程度地检出,含量为2.36~223.30 ng/L。多环芳烃除苊、芴、荧蒽3种未检出外,其他13种PAHs均有检出,含量为0.36~47.63 ng/L,除了苯并[*a*]芘(0.68~4.90 ng/L)超过《地表水环境质量标准》(GB 3838-2002)限值外,其他物质均未超标。国内外一些地区的各类水体中也检测出多种SVOCs,但仍以PAEs、PAHs和OCPs为主^[4,22-24]^。

## 3 结论

本研究建立了液液萃取-气相色谱-质谱同时测定有机氯、多环芳烃、硝基苯、邻苯二甲酸酯等4类共计46种半挥发性有机物的方法。该方法操作简单,准确度及精密度良好,检出限低,可为批量化准确分析地表水及地下水中46种半挥发性有机物的同时检测提供技术参考。
